# In Vivo Molecular Imaging and Histological Analysis of Changes Induced by Electric Pulses Used for Plasmid DNA Electrotransfer to the Skin: A Study in a Dorsal Window Chamber in Mice

**DOI:** 10.1007/s00232-012-9435-5

**Published:** 2012-05-27

**Authors:** Bostjan Markelc, Elisabeth Bellard, Gregor Sersa, Sandrine Pelofy, Justin Teissie, Andrej Coer, Muriel Golzio, Maja Cemazar

**Affiliations:** 1 Department of Experimental Oncology, Institute of Oncology Ljubljana, Zaloska 2, 1000 Ljubljana, Slovenia; 2 IPBS (Institut de Pharmacologie et de Biologie Structurale), CNRS, 205 route de Narbonne, BP 64182, 31077 Toulouse, France; 3 IPBS (Institut de Pharmacologie et de Biologie Structurale), Université de Toulouse, UPS (Université Paul Sabatier), BP 64182, 31077 Toulouse, France; 4 Faculty of Health Sciences, University of Primorska, Polje 42, 6310 Izola, Slovenia

**Keywords:** Electropermeabilization, Electroporation, Blood vessel, Permeability, Vasoconstriction, Vascular lock, Immune cell infiltration, Plasmid DNA

## Abstract

Electropermeabilization/electroporation (EP) is a physical method that by application of electric pulses to cells increases cell membrane permeability and enables the introduction of molecules into the cells. One of the uses of EP in vivo is plasmid DNA electrotransfer to the skin for DNA vaccination. EP of tissues induces reduction of blood flow and, in combination with plasmid DNA, induction of an immune response. One of the EP protocols for plasmid DNA electrotransfer to the skin is a combination of high-voltage (HV) and low-voltage (LV) pulses. However, the effects of this pulse combination on skin-vessel blood flow are not known. Therefore, using intravital microscopy in a dorsal window chamber in mice and fluorescently labeled dextrans, the effects of one HV and eight LV pulses on skin vasculature were investigated. In addition, a detailed histological analysis was performed. Image analysis of fluorescence intensity changes demonstrated that EP induces a transient constriction and increased permeability of blood vessels as well as a “vascular lock.” Histological analysis revealed rounding up of endothelial cells and stacking up of erythrocytes at 1 h after EP. In addition, extravasation of erythrocytes and leukocyte infiltration accompanied by edema were determined up to 24 h after EP. In conclusion, our results show that blood flow modifying effects of EP in skin contribute to the infiltration of immune cells in the exposed area. When combined with plasmid DNA for vaccination, this could enable the initial and prolonged contact of immune cells with encoded therapeutic proteins.

## Introduction

The use of plasmid DNA as a vaccine is a promising alternative to traditional vaccines that are based on live or attenuated viruses (Tang et al. 1992; Rice et al. 2008; Ingolotti et al. 2010; Ferraro et al. 2011). The possibility of delivering a vaccine without inducing antiviral immunity, the ability to formulate multicomponent vaccines (Hirao et al. 2011; Sardesai and Weiner 2011), the stability of DNA at room temperature and the safety of production compared to viral vaccines make plasmid DNA vaccination especially attractive. Proper design of plasmid DNA can lead to enhanced expression of antigen, targeted expression in the cell (cytosol or endoplasmic reticulum) and induction of CD4^+^ T-helper cells, among other things, which leads to an increased immune response (Rice et al. 2008). The initial problems with the delivery of DNA vaccines to cells in vivo and induction of a potent immune response of the host have been successfully surmounted by using electropermeabilization/electroporation (EP) as a delivery system (Drabick et al. 2001; Pavselj and Preat 2005; Rice et al. 2008; Donate et al. 2011; Sardesai and Weiner 2011). EP is a physical method where the application of external electric pulses directly to living cells induces a local increase in transmembrane potential difference. This consequently enables the introduction of molecules into the cells (Miklavcic and Towhidi 2010). The method was first described by Neumann et al. (1982) for the introduction of DNA into cells in vitro; however, clinical use was first achieved for the delivery of cancer chemotherapeutic drugs with EP, such as bleomycin or cisplatin (electrochemotherapy) (Marty et al. 2006; Sersa et al. 2008b). Nowadays, EP is increasingly used for the delivery of different nucleic acids (plasmid DNA, siRNA, miRNA, shRNA, etc.) into cells in vitro and different tissues, including muscle, skin and tumors, in vivo (Rols et al. 1998; Gehl and Mir 1999; Cemazar et al. 2006; Aung et al. 2009; Mir 2009; Escoffre et al. 2010; Vidic et al. 2010). Effective use of EP for enhancement of plasmid DNA vaccination was first reported 10 years ago (Kadowaki et al. 2000; Widera et al. 2000; Drabick et al. 2001) and has already reached the clinical trial stage (van Drunen Littel-van den Hurk and Hannaman 2010; Ferraro et al. 2011; Vasan et al. 2011; El-Kamary et al. 2012). The success of EP for plasmid DNA vaccination was attributed to the fact that EP enhances delivery of plasmid DNA into the cells, where it is expressed for a long time (several months) (Widera et al. 2000; Cemazar et al. 2006; Escoffre et al. 2010), and thus induces a potent immune response of the host to the introduced encoded antigen (Drabick et al. 2001; Liu et al. 2008; Roos et al. 2009; Xing et al. 2012). Furthermore, EP was demonstrated to be safe and well tolerated in preclinical as well as clinical studies (Vanbever and Preat 1999; Sardesai and Weiner 2011; El-Kamary et al. 2012).

The best results were obtained when plasmid DNA vaccination was performed in muscle or skin (Sardesai and Weiner 2011). One of the EP protocols for plasmid DNA electrotransfer used a combination of one short (100 μs) high-voltage (HV) pulse and one or more long (several milliseconds) low-voltage (LV) pulses, the voltage being chosen from the electrode design and the tissue being pulsated. In this setting EP increased DNA expression up to 100-fold in muscle and skin. Also, a higher infiltration of immune cells into the EP area was present, consequently enabling the contact of more antigen-presenting cells (APCs) with encoded antigens (Roos et al. 2009; Tevz et al. 2009; Lee et al. 2011). It was shown that EP induces vascular changes in muscle, which could contribute to the observed immune effects (Gehl et al. 2002). However, the vascular and blood-modifying effects of the HV–LV pulse combination that was used for plasmid DNA electrotransfer to the skin are not known.

Therefore, the aim of the present study was to determine the effects of an HV–LV pulse combination on skin and subcutaneous blood vessels. For this purpose we used in vivo optical imaging in a dorsal window chamber (DWC) (Jain et al. 2002; Dreher et al. 2006; Palmer et al. 2011) in mice together with histological characteristic evaluation: edema, blood-vessel changes and immune cell infiltration at different times after the HV–LV pulse combination.

## Materials and Methods

### Reagents

We resuspended 2,000 kDa fluorescein isothiocyanate (FITC) labeled dextran (Sigma-Aldrich, St. Louis, MO) in phosphate-buffered saline (PBS). To remove any free FITC or low-molecular weight contaminants, the 2,000 kDa FITC-labeled dextran was washed two times for 2 h through a 1,000 kDa Vivaspin ultrafiltration spin column (Sartorius Stedim Biotech, Goettingen, Germany). The high-molecular weight component was then resuspended in PBS to a final concentration of 37.5 mg/ml.

### Animals

In the experiments 6–8 week-old female C57Bl/6 mice weighing 20–24 g were used. Mice were kept under specific pathogen-free conditions at a constant room temperature (21 °C) and humidity and a 12 h light/dark cycle. Food and water were provided ad libitum. Animals were subjected to an adaptation period of 14 days before experiments. All animal experiments were conducted in accordance with the guidelines for animal experiments of the EU directives, the French procedural guidelines for animal handling with the approval of the Regional Ethical Review Committee in Midi-Pyrénées (MP/02/36/10/10) and permission from the Ministry of Agriculture, Forestry and Food of the Republic of Slovenia (permission 34401-12/2009/6). For each experimental condition three to five mice were randomly assigned, out of which two to four mice were selected for histological analysis. Only one experiment was performed on each mouse.

### Preparation of the DWC in Mice

The DWC model in mice is a chronic model where a dorsal skinfold is sandwiched between two symmetrical frames. It allows direct visual access to the normal vasculature of the skin through a standard microscopy cover glass and enables repetitive high-resolution imaging of the exposed vasculature in the same mouse over a period of 2–3 weeks (Palmer et al. 2011). For DWC implantation mice were first anesthetized with an intraperitoneal injection of ketamine (1 mg/ml, Narketan^®^; Vetoquinol, Ittigen, Switzerland), xylazine (5 mg/ml, Chanazine; Chanelle Pharmaceuticals, Loughrea, Ireland) and acepromazine (0.4 mg/ml, Promace; Fort Dodge Animal Health, IA); then, the back of the mouse was shaved and depilated with depilatory cream (Veet; Reckitt Benckiser, Slough, UK). DWC (APJ Trading, Ventura, CA) consisting of two titanium frames was surgically implanted onto the extended double layer of the skin with stainless-steel screws and sutures. Subsequently, one layer of the skin was excised to expose the vasculature of the lower layer of skin. From the lower layer of skin all fat and connective tissues were dissected away to ensure optimal microscopic observation. The DWC was filled with 0.9 % NaCl solution and closed with a 12 mm cover glass (Glaswarenfabrik Karl Hecht, Sondheim, Germany). After the surgery and for the following 2 days, butorphanol (0.3 mg/kg, Torbugesic; Fort Dodge Animal Health) was injected intramuscularly once per day.

### Electropermeabilization

EP was performed 3–7 days after the implantation of DWC in mice. The pulsing parameters used were one HV pulse (voltage-to-distance ratio 1,000 V/cm, duration 100 μs) followed by a 1 s lag and eight LV pulses (voltage-to-distance ratio 140 V/cm, duration 50 ms, repetition frequency 1 Hz) (Andre et al. 2008). Pulses were generated by Cliniporator^™^ (Igea, Carpi, Italy) and delivered by two parallel stainless-steel rods (length 5 mm, width 1.3 mm) 4 mm apart (Mazeres et al. 2009). The electrodes were placed on the skin on the opposite side of the cover glass, where the epidermis was intact. Good contact between the electrodes and the skin was ensured by means of a conductive gel (P. J. Dahlhausen, Cologne, Germany). To determine the leakage of FITC-labeled dextran from the vessels, EP was performed 2 min after FITC-labeled dextran injection, when all vessels were completely filled, or at different times before FITC-labeled dextran injection to determine resealing of the vessel wall and duration of the “vascular lock.”

### Intravital Microscopy and Image Acquisition

For intravital microscopy an upright “Macrofluo” fluorescence microscope (Leica Microsystems, Rueil-Malmaison, France) equipped with a Cool Snap HQ Camera (Roper Scientific, Ottobrunn, Germany) and Metamorph (Molecular Devices, Sunnyvale, CA) image acquisition software were used. Animals were first anesthetized with inhalation anesthesia (Isofluran; Nicholas Piramal India, London, UK) and placed on a custom-designed holder, which enabled fixation of the DWC during image acquisition and therefore prevented the artifacts caused by movement due to breathing. Blood vessels were visualized by fluorescence (excitation filter, BP 480/40 nm; emission filter, LP 510 nm) after injection of 100 μl of FITC-labeled dextran (3.75 mg/mouse). To ensure that the vessels were completely filled up, a series of images every 20 s for 2 min was acquired when FITC-labeled dextran was injected before EP. Immediately after EP (<10 s) a second series of images every 20 s for the first 8 min and every 2 min for the next 22 min was acquired. When FITC-labeled dextran was injected after EP, only the second series of images was acquired. Images were analyzed offline with image analysis software (AxioVision; Zeiss, Jena, Germany).

### Data Analysis

To determine the increase in fluorescence intensity inside and outside the vessels, a differential approach was used, which was adapted from Reyes-Aldasoro et al. (2008), followed by specific image analysis. Briefly, after i.v. injection of FITC-labeled dextran, a series of images was acquired during the first 2 min before EP and 30 min after EP. Then, a mask of the blood-vessel network was created and the mean fluorescence intensity determined in the vascular compartment and in the tissue. For measurement of blood-vessel diameters, at least five venules (diameters 20–250 μm) and arterioles (10–100 μm) were selected from each mouse. The narrow, straight, fast-flowing vessels with few branches were defined as arterioles and the rest as venules. The diameter of the vessels was measured in each image of the recorded series and normalized to the value before EP.

### Histology

The circular part of the skin inside the DWC that was monitored through the cover glass was excised at 1, 12, 24, or 48 h after EP. The skin was then fixed in formalin for 24 h and stored in 70 % ethanol until it was embedded in paraffin in such orientation that sections were cut perpendicular to the skin layers encompassing both parts of the skin where electrodes were placed. From each DWC preparation, 9–12 sections of 5 μm thickness were cut and stained with hematoxylin and eosin. Slides were observed with a BX-51 microscope (Olympus, Hamburg, Germany) coupled with a DP72 digital camera (Olympus).

### Statistical Analysis

For statistical analysis Sigma Plot software (Systat Software, London, UK) was used. For comparison of the control and EP groups, a Student *t*-test or one-way ANOVA followed by a Holm-Sidak test was used. A value of *p* < 0.05 was considered statistically significant.

## Results

### EP Induces Constriction of Blood Vessels

**Fig. 1:**
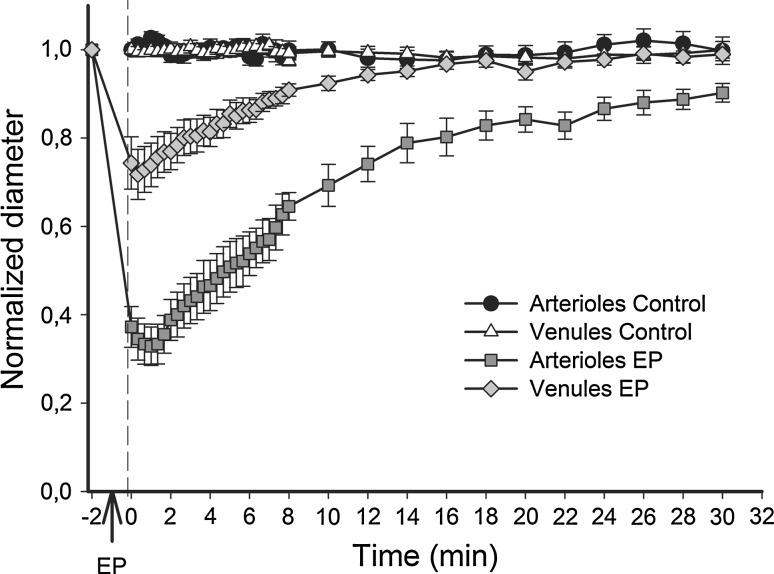
Constriction of blood vessels after EP. Diameters of vessels were measured after i.v. injection of FITC-labeled dextran. Pulsing parameters when EP was applied: one pulse of voltage-to-distance ratio 1,000 V/cm, 100 μs, 1s pause; eight pulses of voltage-to-distance ratio 140 V/cm, 50 ms, 1 Hz. Diameters were normalized to the value 2 min after i.v. injection of FITC-labeled dextran. Normalized diameters of arterioles and venules after EP (*n* = 3) are represented as a function of time in comparison to control (*n* = 3). Diameters were statistically significantly smaller for the first 14 min for venules and throughout the observation time for arterioles (*p* < 0.05). Student’s *t* test was used for comparison of each time point of the EP group to the same time point of the control group

To determine the effect of EP on vessel morphology, the diameters of arterioles and venules were measured for the first 30 min after EP. Application of electric pulses resulted in an immediate constriction of the arterioles and venules (Fig. 1). The statistically significant constriction of arterioles (~70 %) was more pronounced than the constriction of venules (~30 %) and lasted longer (Fig. 1). In comparison to the control, the diameters of arterioles remained statistically significantly smaller during the entire observation period (30 min), whereas the diameters of venules returned to pre-EP values 14 min after EP (Fig. 1). The diameters of arterioles and venules in control mice remained constant during the entire observation time (20–250 μm for venules and 10–100 μm for arterioles).

### EP Induces a “Vascular Lock”

**Fig. 2:**
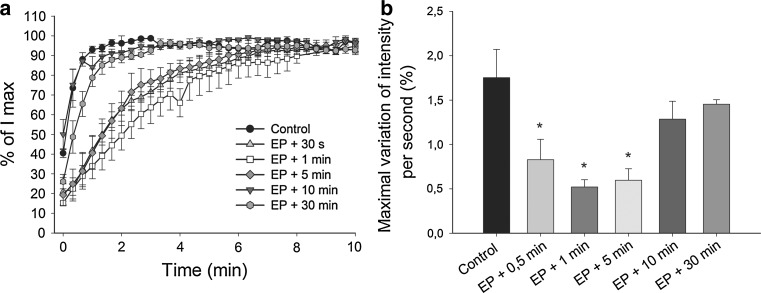
Time course of filling up of blood vessels after EP. Kinetics of filling up of vessels was determined after i.v. injection of FITC-labeled dextran at different times after EP (0.5, 1, 5, 10, and 30 min). Pulsing parameters when EP was applied: one pulse of voltage-to-distance ratio 1,000 V/cm, 100 μs, 1s pause; eight pulses of voltage-to-distance ratio 140 V/cm, 50 ms, 1 Hz. **a** Filling up of vessels is expressed as a percent of maximum mean fluorescence intensity in vessels (*I*_max_) after i.v. injection of FITC-labeled dextran. **b** Maximal variation of intensity per second calculated from the filling up curves. The filling up of vessels was slowed down when dextran was i.v. injected 0.5, 1, and 5 min after EP and returned to control levels when the interval between EP and i.v. -injection was 10 and 30 min (*n* = 3–5). A one-way ANOVA followed by a Holm-Sidak test were used for statistical analysis. *p < 0.05 in comparison to the control. *Error bars* indicate SEM

**Fig. 3:**
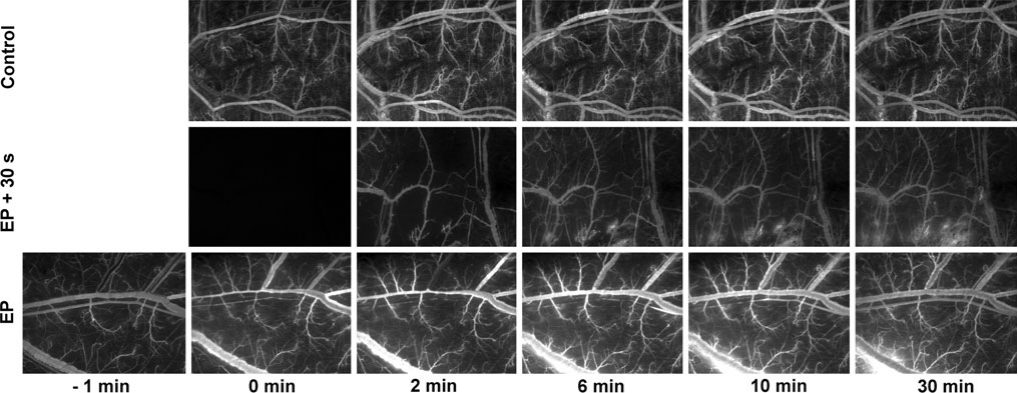
Illustration of “vascular lock” and dextran leakage into the tissue after EP. Pulsing parameters when EP was applied: one pulse of voltage-to-distance ratio 1,000 V/cm, 100 μs, 1s pause; eight pulses of voltage-to-distance ratio 140 V/cm, 50 ms, 1 Hz. “Vascular lock” was determined when FITC-labeled dextran was i.v. -injected 0.5 min after EP (*EP* *+* *30* *s*), where the filling up of vessels was completed ~10 min after EP, whereas leakage of FITC-labeled dextran was observed earlier (~6 min after EP). Leakage of FITC-labeled dextran into the tissue was measured when FITC-labeled dextran was i.v. -injected 2 min before EP (*EP*), and then relative variation of mean fluorescence intensity was determined for the next 30 min. In the control group (*Control*) filling up of vessels was completed within 2 min after i.v. injection of FITC-labeled dextran, and there was no leakage of dextran into the tissue

EP induced transient obstruction of blood flow, a “vascular lock” in the skin, which resulted in slowed filling up of vessels in comparison to the control group, where complete filling up of vessels was achieved within 2 min after FITC-labeled dextran i.v. injection (Fig. 2a). When FITC-labeled dextran was injected 0.5, 1, and 5 min after EP, the filling up of all pulsed vessels was completed ~10 min after i.v. injection (Figs. 2,3). When FITC-labeled dextran was injected 10 or 30 min after EP, the filling up of vessels was completed within 3 min after i.v. injection (Fig. 2). The maximal variation of mean fluorescence intensity per second was also statistically significantly decreased at 0.5, 1, and 5 min after EP (Fig. 2b). This demonstrates that the EP-induced “vascular lock” in the skin is transient and lasts ~10 min after EP.

### EP Increases the Permeability of Blood Vessels

**Fig. 4:**
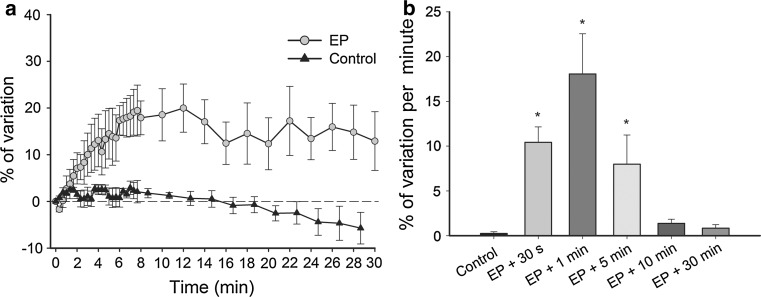
Increased permeability of blood vessels and duration of increased permeability. Pulsing parameters when EP was applied: one pulse of voltage-to-distance ratio 1,000 V/cm, 100 μs, 1s pause; eight pulses of voltage-to-distance ratio 140 V/cm, 50 ms, 1 Hz. **a** Measure of relative mean fluorescence intensity changes in DWC tissues outside the vessels as a function of time after EP in comparison to the control group without EP. **b** Measure of relative mean fluorescence intensity changes per minute in DWC tissues outside the vessels when FITC-labeled dextran was injected at different times after EP (*n* = 3–5). A one-way ANOVA followed by a Holm-Sidak test were used for statistical analysis. *p < 0.05 in comparison to control. *Error bars* indicate SEM

To determine the effect of EP on the permeability of blood vessels, the relative variation of the mean fluorescence intensity in the tissue neighboring the blood vessel due to FITC-labeled dextran leakage from the pulsed blood vessels was measured (Fig. 3). A statistically significant increase of the mean fluorescence intensity was determined after EP in comparison to the control. For the first 8 min after EP, the increase of relative variation of fluorescence intensity was linearly dependent on time, after which it reached a plateau (Fig. 4a). In the control group the relative variation of mean fluorescence intensity in the tissue decreased slightly during the observation time (Fig. 4a).

To evaluate the duration of increased permeability of the pulsed blood vessels, FITC-labeled dextran was i.v.-injected at different times after EP and the relative increase of mean fluorescence in the tissue per minute was determined thereafter (Figs. 3, 4b). Statistically significant values were determined for the injection of FITC-labeled dextran 0.5, 1, and 5 min after EP; then, the relative variation of mean fluorescence intensity per minute decreased and reached values similar to the control group when injection of FITC-labeled dextran was performed 30 min after EP (Fig. 4b). For the calculations, only the first 8 min after FITC-labeled dextran injection were relevant due to the fact that during this time interval after EP the FITC-labeled dextran leakage from blood vessels into the tissue was linearly dependent on time (Fig. 4a).

### EP-Induced Histological Changes of the Skin

**Fig. 5:**
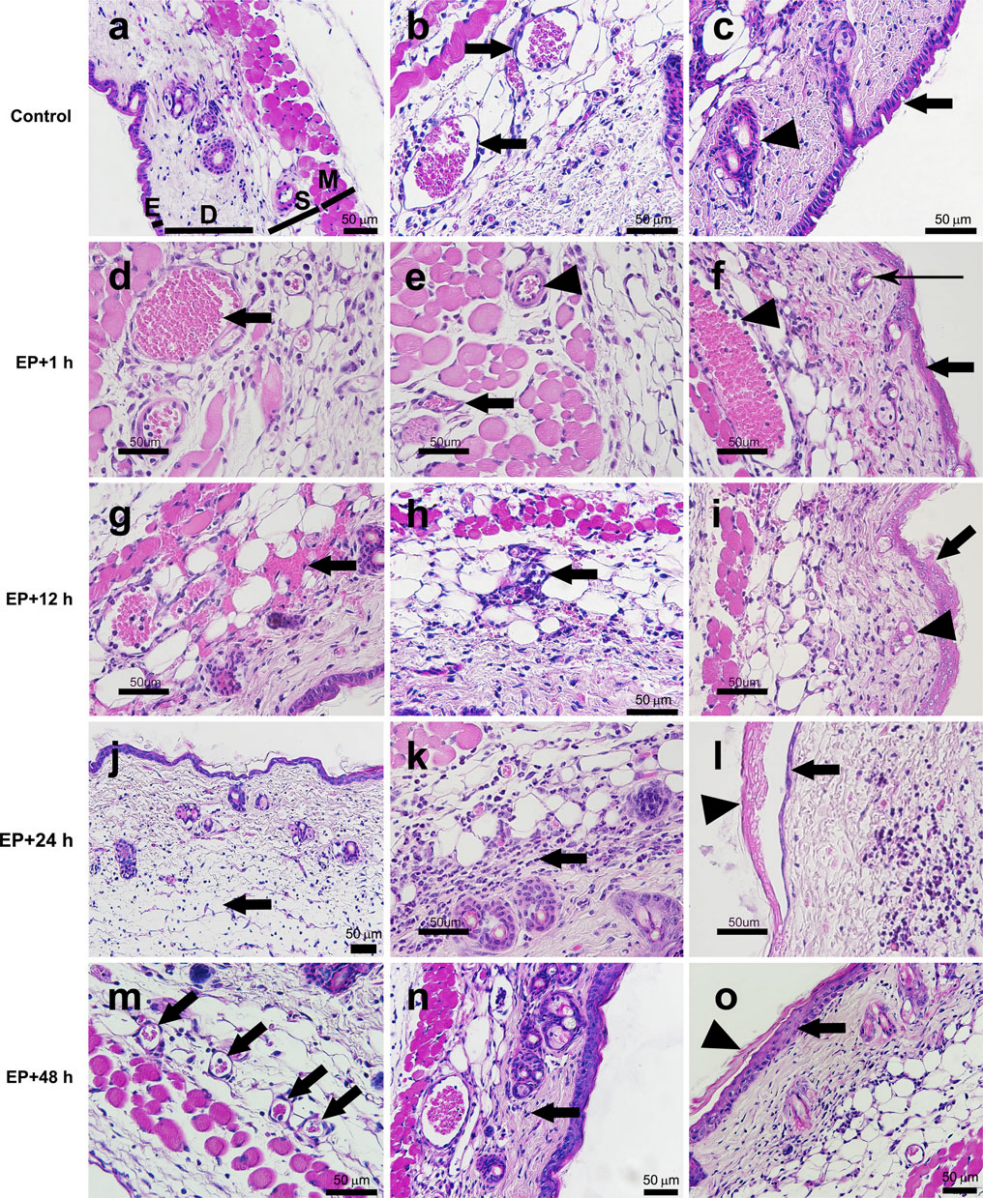
EP-induced changes in skin. Representative images of tissue sections which were exposed to EP and stained with hematoxylin and eosin (pulsing parameters: one pulse of voltage-to-distance ratio 1,000 V/cm, 100 μs, 1s pause; eight pulses of voltage-to-distance ratio 140 V/cm, 50 ms, 1 Hz). **a** *E* epidermis, *D* dermis with hair follicles, *S* subcutaneous tissues with blood vessels, *M* a thin striated muscle layer; **b** *arrow* blood vessels; **c** *arrow* intact epidermis, *arrowhead* hair follicle; **d** *arrow* stacking of erythrocytes; **e** *arrow* rounding up of endothelial cells in the venule, *arrowhead* rounding up of endothelial cells in the arteriole; **f** *thick arrow* damaged keratinocytes under the electrode, *arrowhead* marginalization of immune cells (prominent nuclei are present) in the blood vessel, *thin arrow* damaged hair follicle; **g** *arrow* extravasation of erythrocytes; **h** *arrow* infiltration of immune cells; **i** *arrow* damaged keratinocytes under the electrode, *arrowhead* damaged hair follicle; **j** *arrow* edema; **k** *arrow* infiltration of immune cells; **l** *arrow* recovery of epidermis, *arrowhead* damaged keratinocytes under the electrode; **m** *arrow* recovered/normal blood vessels; **n** *arrow* infiltration of immune cells; **o** *arrow* recovery of epidermis, *arrowhead* damaged keratinocytes under the electrode. *Scale bar* 50 μm

Histological changes of the skin after EP were determined 1, 12, 24, and 48 h after EP. In the slides, sections of the skin consisting of epidermis, dermis with hair follicles, subcutaneous tissues with blood vessels and a thin striated muscle layer are presented (Fig. 5a). Rounding up of vessel endothelial cells, narrowing of vessel lumen and stacking of erythrocytes in vessels were present at 1 h after EP (Fig. 5d–f) but were not present anymore at later time points. The extravasation of erythrocytes; increased presence and marginalization of leukocytes, which was accompanied by extravasation; and increased infiltration of leukocytes into the tissue between the electrodes was observed at 1 and 12 h after EP (Fig. 5d–i). Infiltration of leukocytes was also observed at 24 h after EP, which decreased at 48 h after EP (Fig. 5j–o). Infiltration was accompanied by edema, which was present in the connective tissue under the epidermis at all time points, except at 48 h after EP, when it was already resolved (Fig. 5). All of these features were more pronounced closer to the negative electrode than under the positive one or between the electrodes. In addition, at all time points the damage to the epidermis, vessels and hair follicles was much more evident under the negative electrode, where even destruction of different tissue structures was observed (Fig. 5). Recovery of the epidermis and underlying tissue under the electrodes was evident at 24 and 48 h after EP (Fig. 5g–o). All of the above-mentioned features were observed in all samples at specific time points.

## Discussion

The results of this study show that application of an HV–LV pulse combination used for plasmid DNA electrotransfer to the skin induces a transient constriction of blood vessels, “vascular lock” and increased permeability of blood vessels. Already within 1 h after EP rounding up of endothelial cells and stacking up of erythrocytes can be observed in the area of the tissue exposed to EP. In addition, extravasation of erythrocytes into the tissue and leukocyte infiltration with edema were observed. Damage to the epidermis and the tissue beneath the electrodes was repaired within 48 h after EP.

Several studies have reported that different HV–LV combinations of pulses are very effective for plasmid DNA electrotransfer to the skin, inducing a better immune response and longer gene expression in comparison to HV or LV pulses alone (Pavselj and Preat 2005; Andre et al. 2008; Roos et al. 2009; Brave et al. 2010; Gothelf et al. 2011). Concerning the safety of the methods, regardless of the electrodes used, the application of pulses to the skin results in a slight and transient disruption of the skin barrier function, slight and transient erythema and upregulation of genes involved in the immune response (Dujardin et al. 2002; Pavselj and Preat 2005; Roos et al. 2009). However, there have been no reports on the effects of EP on the vasculature of the skin and detailed histological analysis of these effects. Our study is the first to provide direct observations of the effects of an HV–LV combination of pulses on normal vasculature in the skin.

First, the effects of EP on blood vessels in the skin from our direct observation extended the models of vascular response observed in studies done on muscle and tumors (Gehl et al. 2002; Sersa et al. 2008a). The vascular response was described as a two-phase phenomenon, where the first short phase is quick and was attributed to sympathetically mediated vasoconstriction of afferent arterioles, whereas the second slower phase is of much longer duration (up to 30 min in the case of muscle) and supposed to follow the kinetics of cell membrane resealing after EP and changes of endothelial cell shape (Gehl et al. 2002; Jarm et al. 2010). Our data on measurement of blood-vessel diameters are in agreement with this hypothesis. The immediate constriction of blood vessels after EP was more pronounced for arterioles (~70 %) than for venules (~30 %). The constriction of arterioles also lasted longer (throughout the observation period) than the constriction of venules (~15 min). The bigger impact of EP on the diameter of arterioles can be explained by differences in the structure of the blood-vessel wall, where the smooth muscle of the tunica media is more abundant in the wall of arterioles compared to venules (Seeley et al. 2000). The observed constriction of the blood vessels resulted in a “vascular lock” (Gehl et al. 2002; Sersa et al. 2008a) (Fig. 2). The filling up of the vessels was statistically significantly slower for the first 5 min after EP. For example, when FITC-labeled dextran was injected immediately after EP (0.5 min), the filling up of the blood vessels was completed ~10 min after the injection. Direct in vivo experimental support for the second phase of the proposed vascular effects was demonstrated in our study by increased permeability of the blood vessels after EP for 2,000 kDa FITC-labeled dextran. Its size was chosen as being approximately the size of plasmid DNA (Paganin-Gioanni et al. 2011). The increased permeability of the blood vessels for 2,000 kDa FITC-labeled dextran lasted for 5–10 min and gradually returned to pre-EP values 30 min after EP. The second phase was mainly attributed to disruption of the cellular cytoskeleton and junctional integrity of the blood-vessel endothelium (Kanthou et al. 2006; Jarm et al. 2010). The increased permeability of the blood vessels for 2,000 kDa FITC-labeled dextran returned to control levels only 30 min after EP, indicating that restoration of the blood-vessel endothelium is slow and, thus, enables leakage of large molecules for an extended period of time.

Second, EP also has other effects on the target tissue besides vascular effects, and very little is known about these effects. It is now known that application of electric pulses to tissues affects not only the permeability of cell membranes but also the distribution of DNA and other molecules in the tissues (Zaharoff et al. 2002; Henshaw et al. 2007, 2011). One of the hallmarks of DNA vaccination combined with EP is that EP significantly enhances the immunogenicity of DNA vaccines relative to DNA injection alone also in human clinical trials (Hirao et al. 2010; Livingston et al. 2010; Sardesai and Weiner 2011; Vasan et al. 2011). This was concluded by measuring different features of the immune response, but the underlying mechanisms remain poorly understood. DNA vaccination, regardless of the therapeutic gene used, combined with EP increased the excretion of interferon-gamma from antigen-specific CD8^+^ cells, increased the number of antigen-specific CD4^+^ cells and several other types of immune cells compared to injection of DNA alone or another type of vaccination (Rosati et al. 2009; Belisle et al. 2011; Brave et al. 2011; Kulkarni et al. 2011; Lee et al. 2011; Sardesai and Weiner 2011). The results of our study demonstrate that the effects of EP on skin blood vessels can contribute to the immune response observed in other studies when EP was combined with DNA vaccines. Namely, the observed effects of EP, i.e., increased permeability of blood vessels and “vascular lock,” cause a small and reversible tissue damage that is enough to lead to inflammation and increased infiltration of immune cells. Specifically, EP induces increased permeability of the blood vessels for at least 30 min, which enables protein leakage outside the vessels, leading to a decrease in the pressure between the intra- and extra-vascular compartments and to extravasation of fluids. The results are development of interstitial edema, increased interstitial fluid pressure and decreased intravascular pressure, which all contribute to compromised blood flow, i.e. “vascular lock,” in addition to vasoconstriction. Furthermore, although the observed “vascular lock” was short-lived, it can still result in ischemia–reperfusion injury (van den Heuvel et al. 2009). Moreover, it was shown in our previous studies that EP causes the formation of reactive oxygen species in vitro, which was indirectly shown also in vivo in mouse muscle (Gabriel and Teissie 1995; Markelc et al. 2012). All of these effects of EP on skin and subcutaneous vasculature observed by in vivo imaging were confirmed by histological analysis performed at different times after EP. Increased permeability of the blood vessels was demonstrated by rounding up of endothelial cells, extravasation of erythrocytes and edema present in the connective tissue below the epidermis. Furthermore, attraction of immune cells was demonstrated by the marginalization of leukocytes in the vessels and by pronounced infiltration seen only in the areas between the electrodes, which were present for at least 24 h. Therefore, taken collectively, in case of plasmid DNA vaccine combined with EP, we can predict that the cells transfected with plasmid DNA are in contact with numerous immune cells for at least 24 h. Thus, the plasmid DNA has enough time to translate into an antigenic protein, which can react with immune cells, leading to a pronounced immune response, both cellular and humoral. It is also worth mentioning that the effects of EP on histological changes (tissue damage, lymphocyte infiltration, etc.) were more pronounced at the negative electrode. The reason for this observation is not known, but it could be ascribed to the electrolytic chemical reaction of reduction at the negative electrode, leading to formation of different chemicals, mainly sodium hydroxide (Wagner et al. 1985).

One of the aspects dealing with translation of plasmid DNA vaccination combined with EP to more widespread use in humans is the standardization of EP parameters. From the tissue effects of EP that were shown in previous studies in muscle, tumors and our study on the skin, it is obvious that the EP parameters optimized for therapeutic effects are dependent on the tissue’s structural characteristics and blood-vessel morphology. Therefore, further studies are needed to evaluate the tissue effect of EP, which will bring plasmid DNA vaccination combined with EP into wider clinical use.
